# Supplementary material for: Mitochondrial network adaptations of microglia reveal sex-specific stress response after injury and UCP2 knockout
Source: iScience. 2023 Aug 29;26(10):107780. doi: 10.1016/j.isci.2023.107780 (PMC10507162; doi:10.1016/j.isci.2023.107780)
Supplement: Document S1. Figures S1–S5 and Table S11 [file mmc1.pdf]

iScience, Volume *26*

Supplemental information

Mitochondrial network adaptations of microglia reveal sex-specific stress response after injury and UCP2 knockout

Margaret E. Maes, Gloria Colombo, Florianne E. Schoot Uiterkamp, Felix Sternberg, Alessandro Venturino, Elena E. Pohl, and Sandra Siegert

Figure S1.

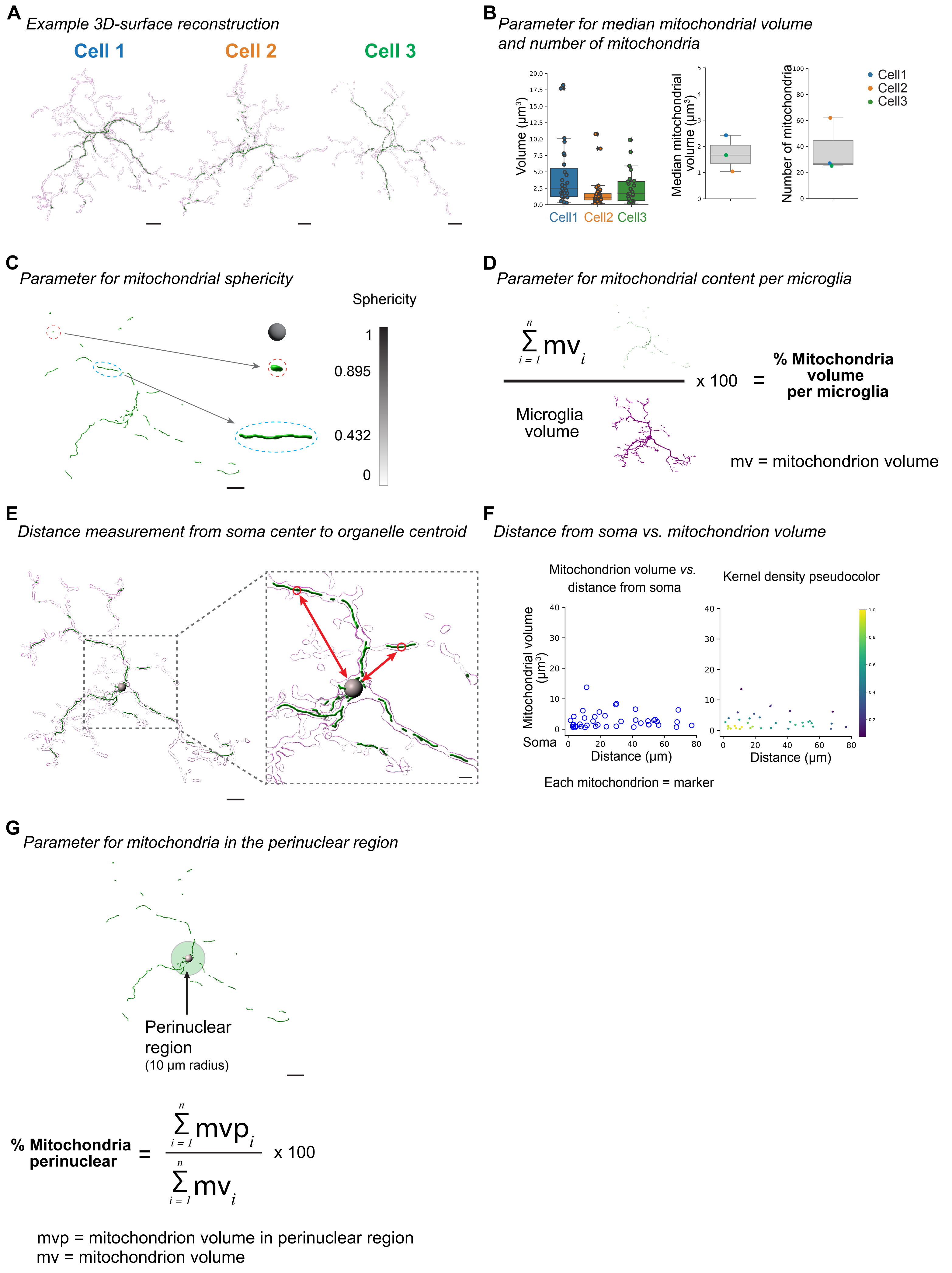
A
Example 3D-surface reconstruction
Cell 1
Cell 2
Cell 3
B
Parameter for median mitochondrial volume and number of mitochondria
Volume (μm³)
Cell1
Cell2
Cell3
Median mitochondrial volume (μm³)
Number of mitochondria
C
Parameter for mitochondrial sphericity
Sphericity
1
0.895
0.432
0
D
Parameter for mitochondrial content per microglia
Microglia volume
x 100
=
% Mitochondria volume per microglia
mv = mitochondrion volume
E
Distance measurement from soma center to organelle centroid
F
Distance from soma vs. mitochondrion volume
Mitochondrion volume vs. distance from soma
Kernel density pseudocolor
Mitochondrial volume (μm³)
Soma
Distance (μm)
Each mitochondrion = marker
G
Parameter for mitochondria in the perinuclear region
Perinuclear region
(10 μm radius)
% Mitochondria perinuclear
x 100
mvp = mitochondrion volume in perinuclear region
mv = mitochondrion volume

Figure S1. Analyzed mitochondrial parameters in microglia, Related to Figure 1

Introduction to the mitochondrial parameters based on three 3D-surface reconstructions of IBA1-immunostained microglia (magenta) from IPL WT (magenta) expressing mito-Dendra2 (green, **A**). Scale bar: 10 µm.

(**B**) Left, boxplot for each cell indicating the volume of every mitochondrial organelle per cell. Each dot, a mitochondrion. Center, median mitochondrial volume and right, number of mitochondria for each cell at left. Boxplot minimum and maximum: InterQuartile Range (IQR) around median (center line). Whiskers: 1.5 IQRs. Black diamond points: outliers outside 1.5 IQRs.

(**C**) Mitochondrial sphericity. Left, example mitochondrial 3D-surface (green) from a WT microglia. Ratio of mitochondrion volume to mitochondrion surface area represents sphericity with two exemplar organelles (orange, sphericity =0.895) and elongated (blue, sphericity = 0.432). Sphericity is represented as a mean sphericity value of all mitochondria per cell. Scale bar: 15 µm.

(**D**) Percentage of the sum of mitochondrial volume in a cell (**Figure S1B, left**) over the microglial volume represents mitochondrial content per cell.

(**E**) Vesicle distance measurement. Example 3D-volume surfaces of an IBA1-immunostained microglia (magenta) with mito-Dendra2 expression (green). Grey sphere: cell soma. Right, zoom-in of region of interest (dashed line). Example red circles: centroid of mitochondrion. Arrows: distance from centroid to soma determined from the centroid of each mitochondrion/vesicle to the center of the soma-sphere (red arrows). Scale bar: 15 µm, zoom-in: 5 µm.

(**F**) Mitochondria or CD68 vesicle localization parameter exemplified using mitochondrial localization. Scatterplot depicts the distance from the soma (µm) *vs.* the mitochondrial volume (µm^3) for each mitochondrion shown in (**Figure S1E**). To illustrate overlapping mitochondria, scatterplot markers were pseudo-colored with kernel density estimations.

(**G**) Example mitochondrial surface (green) from microglia depicted in (**Figure S1E**). The perinuclear region (shaded green sphere) was determined as the region within a 10 µm radius from the cell soma point. The percentage of mitochondria in the perinuclear region is the sum volume within the perinuclear region over the total mitochondrial volume. Scale bar: 15 µm.

Figure S2.

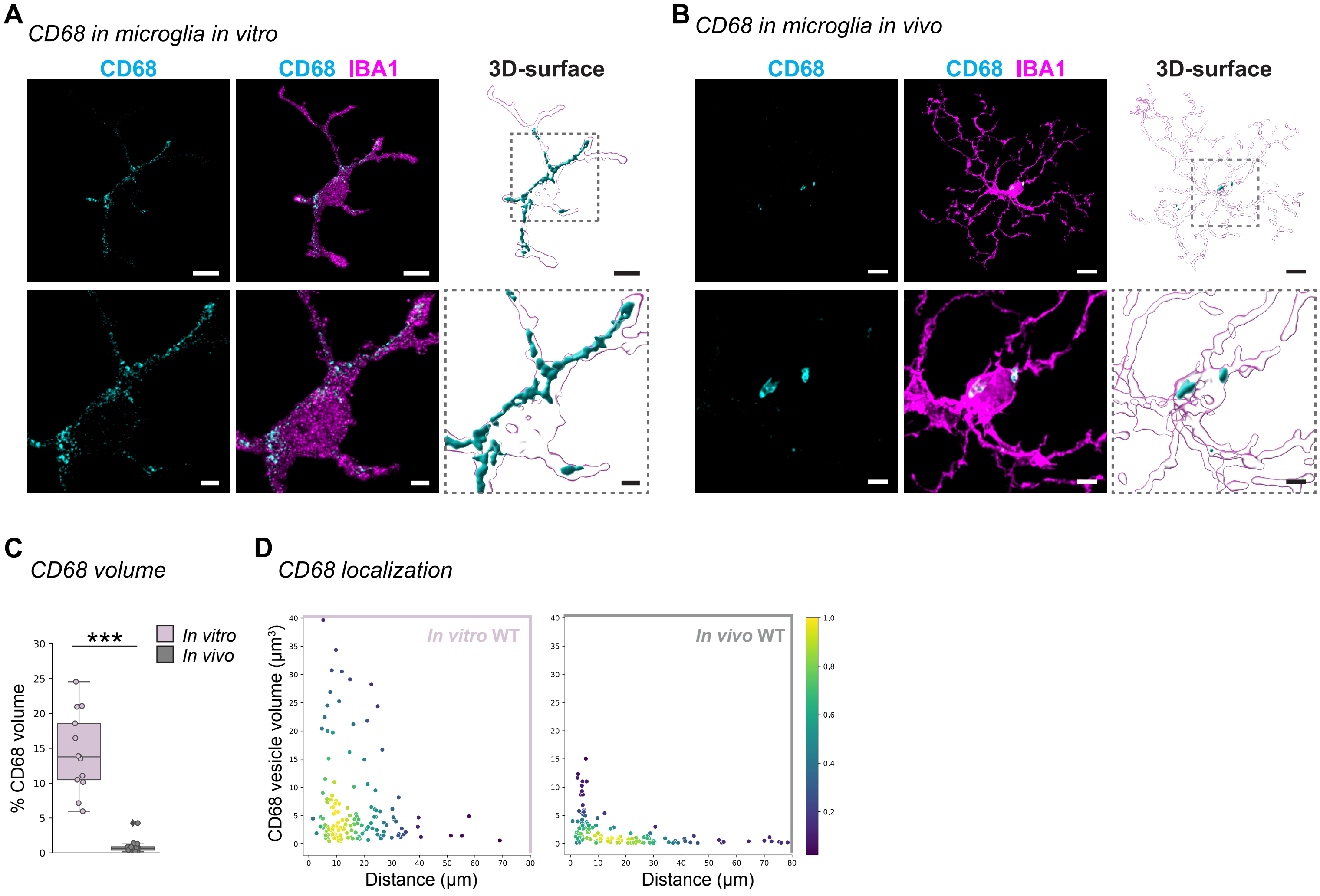
A
CD68 in microglia in vitro
CD68
CD68 IBA1
3D-surface
B
CD68 in microglia in vivo
CD68
CD68 IBA1
3D-surface
C
CD68 volume
***
In vitro
In vivo
% CD68 volume
0
5
10
15
20
25
30
D
CD68 localization
In vitro WT
In vivo WT
CD68 vesicle volume (μm³)
Distance (μm)
Distance (μm)
0
10
20
30
40
50
60
70
80
0
5
10
15
20
25
30
35
40
0.2
0.4
0.6
0.8
1.0

Figure S2. Microglia *in vitro* exhibit increased CD68 expression, Related to Figure 1

(**A**-**B**) IBA1-immunostained microglia (magenta) from **Figure 1F**-**G** co-labeled with CD68 (cyan) from *in vitro* (**A**) or *in vivo* microglia (**B**). Next, corresponding 3D-surfaces. Below: Zoom-in of region of interest (dashed outline) from image and 3D-surface. Scale bar: 10 µm, zoom-in: 3 µm.

(**C**) Percentage of total CD68 volume per microglia volume. Boxplot minimum and maximum: InterQuartile Range (IQR) around median (center line). Whiskers: 1.5 IQRs. Black diamond points: outliers outside 1.5 IQRs. Wilcoxon rank sum test: $p < 0.0001$.

(**D**) CD68 vesicle localization. Scatterplot depicting CD68 vesicle volume *vs.* distance from the cell soma (0, origin) of the population of vesicles in *in vitro* (left, dusty pink) and *in vivo* (right, grey) conditions. Point density, pseudo-colored.

*** $p < 0.001$. See **Table S7** for experimental, retina and cell numbers, statistical tests and corresponding data.

Figure S3.

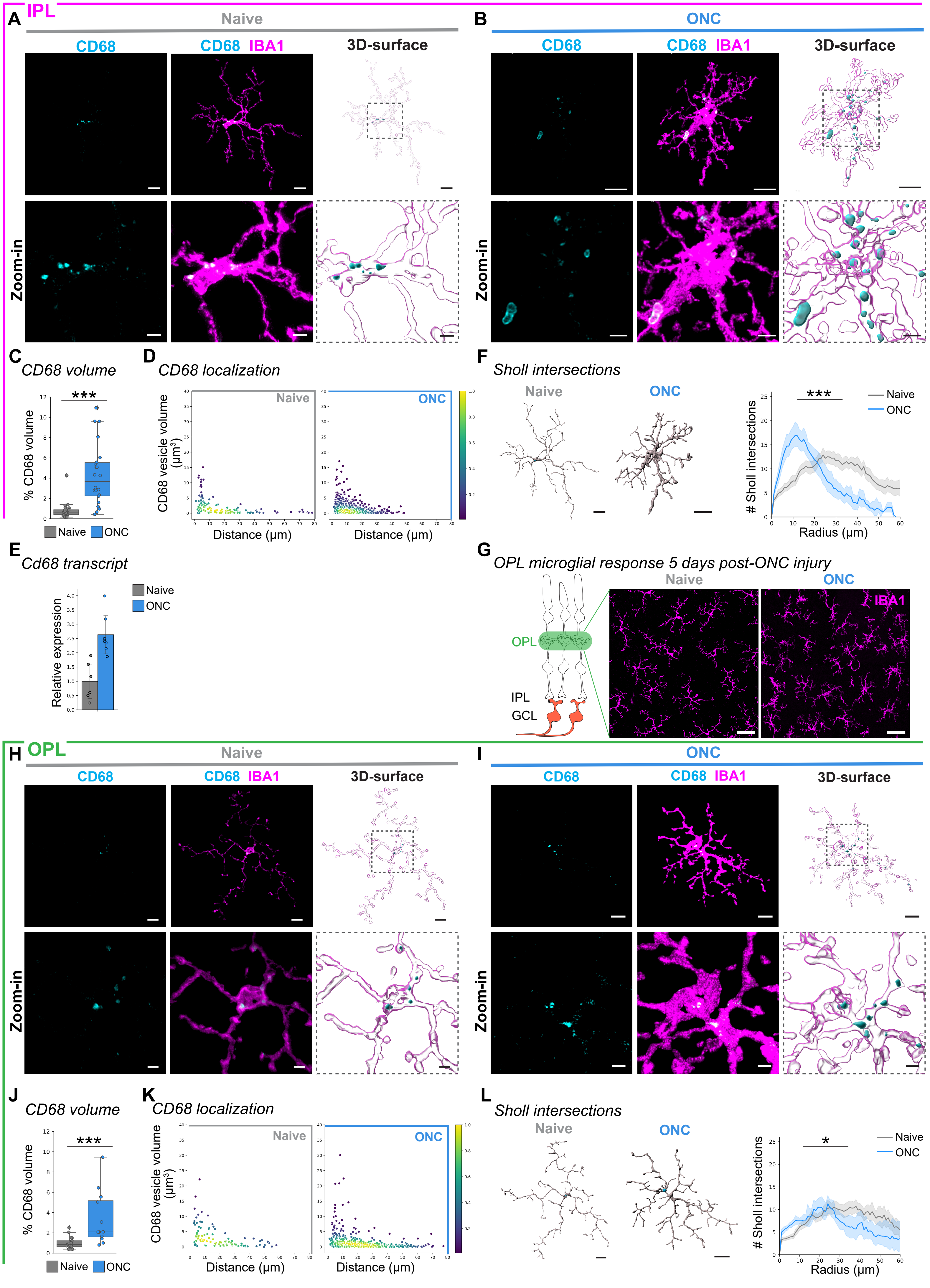
A IPL
Naive
CD68
CD68 IBA1
3D-surface
Zoom-in
B
ONC
CD68
CD68 IBA1
3D-surface
Zoom-in
C CD68 volume
% CD68 volume
***
Naive ONC
D CD68 localization
CD68 vesicle volume (μm³)
Naive
ONC
Distance (μm)
Distance (μm)
F Sholl intersections
Naive
ONC
Sholl intersections
***
Naive
ONC
Radius (μm)
E Cd68 transcript
Relative expression
Naive
ONC
G OPL microglial response 5 days post-ONC injury
Naive
ONC
IBA1
OPL
IPL
GCL
H OPL
Naive
CD68
CD68 IBA1
3D-surface
Zoom-in
I
ONC
CD68
CD68 IBA1
3D-surface
Zoom-in
J CD68 volume
% CD68 volume
***
Naive ONC
K CD68 localization
CD68 vesicle volume (μm³)
Naive
ONC
Distance (μm)
Distance (μm)
L Sholl intersections
Naive
ONC
Sholl intersections
*
Naive
ONC
Radius (μm)

Figure S3. CD68 and microglial morphology changes in ONC-responsive IPL and OPL microglia, Related to Figure 2

(**A-D, F**) Microglial response in IPL WT microglia.

(**A-B**) Representative IBA1-immunostained IPL microglia (magenta) from **Figure 2D-E** co-labeled with CD68 (cyan) in naïve WT (**A**, grey) or ONC WT microglia (**B**, blue). Next, corresponding 3D-surfaces. Below: Zoom-in of region of interest (dashed line) from image and 3D-surface. Scale bar: 10 µm, zoom-in: 3 µm.

(**C**) Percentage of total CD68 volume per microglia volume. Boxplot minimum and maximum: InterQuartile Range (IQR) around median (center line). Whiskers: 1.5 IQRs. Black diamond points: outliers outside 1.5 IQRs. Wilcoxon rank sum test: $p < 0.0001$.

(**D**) CD68 vesicle localization. Scatterplot depicting CD68 vesicle volume *vs.* distance from the cell soma (0, origin) of the population of vesicles in naïve WT (left) and ONC WT (right) conditions. Point density, pseudo-colored.

(**E**) Bar plot depicting relative *Cd68* transcript expression from FACSed mito-Dendra2$^+$ microglia in naïve WT or ONC WT retinas. Welch's t-test: $p = 0.0137$.

(**F**) 3D-filament tracings of microglia from naïve WT (left) or ONC WT (right). Scale bar: 10 µm. Line plot for mean number of Sholl intersections per radial distant from the soma (µm) with 95% confidence interval band. Linear mixed effects model: $p < 0.0001$.

(**G**) Left, retinal side-view schematic. OPL, outer plexiform layer. IPL, inner plexiform layer. GCL, ganglion cell layer. Overview confocal image of immunostained OPL microglia (IBA1, magenta) in naïve WT (left) and 5 days after ONC WT (right) condition. Scale bar: 30 µm.

(**H-L**) Microglial response in OPL WT microglia.

(**H-I**) CD68 (cyan) immunostaining in the IBA1-immunostained OPL microglia (magenta) from **Figure 2I-J** for naïve WT microglia (**H**) or ONC WT microglia (**I**). Next, corresponding 3D-surfaces. Below: Zoom-in of region of interest (dashed outline) from image and 3D-surface. Scale bar: 10 µm, zoom-in: 3 µm.

(**J**) Percentage of total CD68 volume per microglia volume. Boxplot minimum and maximum: InterQuartile Range (IQR) around median (center line). Whiskers: 1.5 IQRs. Black diamond points: outliers outside 1.5 IQRs. Wilcoxon rank sum test: $p = 0.0007$.

(**K**) CD68 vesicle localization. Scatterplot depicting CD68 vesicle volume *vs.* distance from the cell soma (0, origin) of the population of vesicles in naïve (left, grey) and ONC (right, blue) conditions. Point density, pseudo-colored.

(**L**) 3D-filament tracings of microglia from naïve WT (left) or ONC WT (right). Scale bar: 10 µm. Line plot for mean number of Sholl intersections per radial distant from the soma (µm) with 95% confidence interval band. Linear mixed effects model: $p = 0.0302$.

* $p < 0.05$, *** $p < 0.001$. See **Table S8** for retina and cell numbers, statistical tests and corresponding data.

Figure S4.

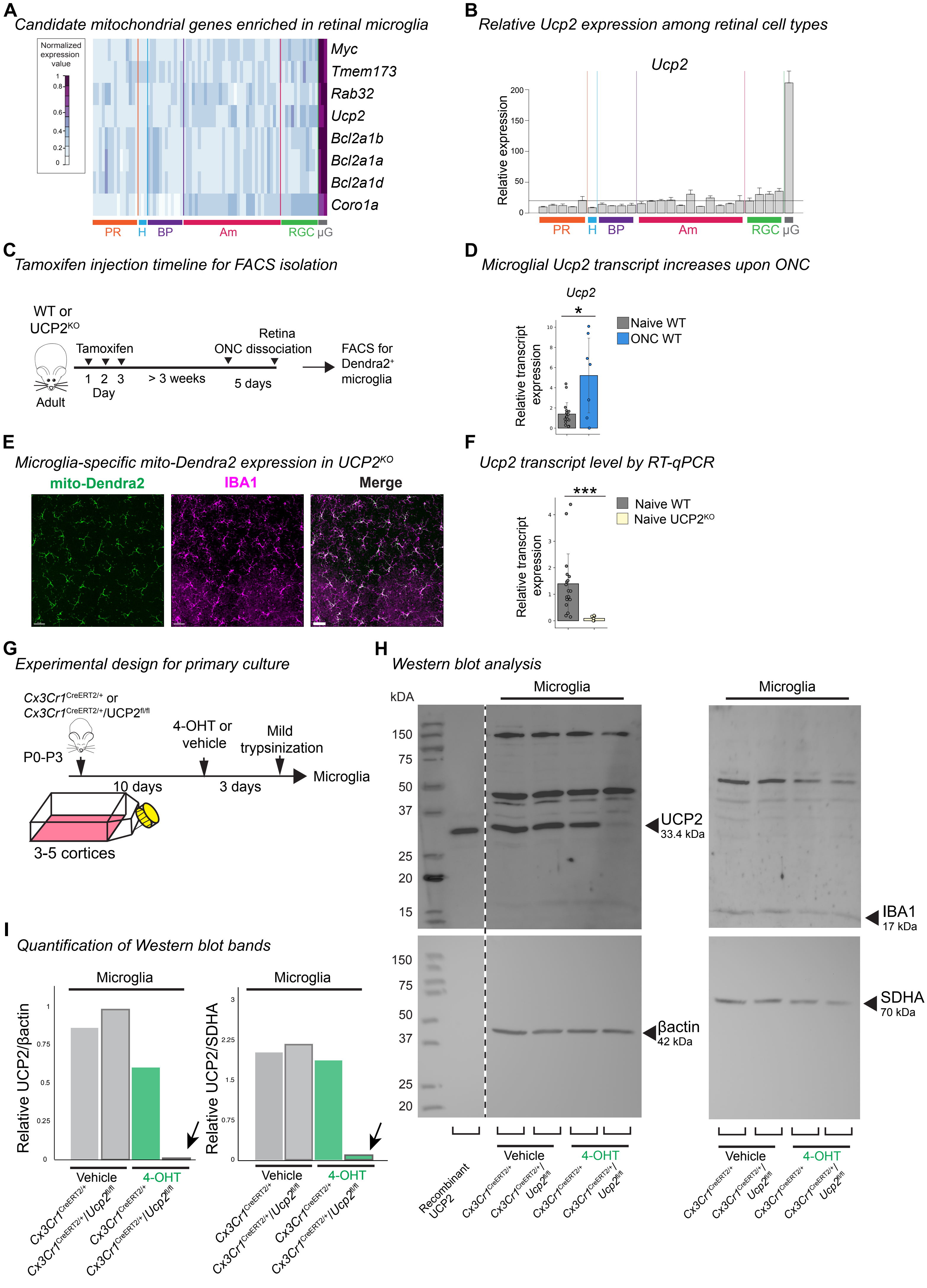

A
Candidate mitochondrial genes enriched in retinal microglia
Normalized expression value
Myc
Tmem173
Rab32
Ucp2
Bcl2a1b
Bcl2a1a
Bcl2a1d
Coro1a
PR H BP Am RGC μG
B
Relative Ucp2 expression among retinal cell types
Ucp2
Relative expression
PR H BP Am RGC μG
C
Tamoxifen injection timeline for FACS isolation
WT or UCP2KO
Adult
Tamoxifen
1 2 3
Day
> 3 weeks
ONC
Retina dissociation
5 days
FACS for Dendra2+ microglia
D
Microglial Ucp2 transcript increases upon ONC
Ucp2
Relative transcript expression
Naive WT
ONC WT
E
Microglia-specific mito-Dendra2 expression in UCP2KO
mito-Dendra2
IBA1
Merge
F
Ucp2 transcript level by RT-qPCR
Relative transcript expression
Naive WT
Naive UCP2KO
G
Experimental design for primary culture
Cx3Cr1CreERT2/+ or Cx3Cr1CreERT2/+/UCP2fl/fl
P0-P3
10 days
4-OHT or vehicle
3 days
Mild trypsinization
Microglia
3-5 cortices
H
Western blot analysis
kDA
Microglia
UCP2 33.4 kDa
βactin 42 kDa
IBA1 17 kDa
SDHA 70 kDa
Recombinant UCP2
Vehicle
4-OHT
Cx3Cr1CreERT2/+
Cx3Cr1CreERT2/+/Ucp2fl/fl
I
Quantification of Western blot bands
Microglia
Relative UCP2/βactin
Relative UCP2/SDHA
Vehicle
4-OHT
Cx3Cr1CreERT2/+
Cx3Cr1CreERT2/+/Ucp2fl/fl

Figure S4. Selective expression and knockout of UCP2 in retinal microglia, Related to Figure 3

(**A**-**B**) Relative fold change expression level of mitochondrial-associated genes (**A**) and *Ucp2* (**B**) among retinal cell types. Plotted from available data in *Siegert et. al*. (1). Purple and blue: high and low normalized expression value, respectively. PR: photoreceptors, H: horizontal cells, BP: bipolar cells, Am: amacrine cells, RGC: retinal ganglion cells, µG: microglia.

(**C**) Timeline for tamoxifen administration to induce mito-Dendra2 expression or in combination $UCP2^{KO}$ in microglia used for FACS (fluorescence activated cell sorting).

(**D**) Boxplot of relative *Ucp2* transcript expression from $Dendra2^{+}$-FACSed retinal microglia in naïve WT or 5 days ONC WT. Boxplot minimum and maximum: InterQuartile Range (IQR) around median (center line). Whiskers: 1.5 IQRs. Black diamond: outliers outside of 1.5 IQRs. Naïve: grey. ONC: blue. Wilcoxon rank sum test: $p = 0.0474$.

(**E**) Microglial specificity of mito-Dendra2 labeling in $UCP2^{KO}$ mouse model. Overview image of immunostained microglia (IBA1, magenta) in the inner plexiform layer (IPL) expressing the mitochondrial label (Dendra2, green) in retinal wholemounts. Scale bar: 50 µm.

(**F**) Relative *Ucp2* transcript expression from tamoxifen-induced naïve WT or naïve $UCP2^{KO}$ FACSed-microglia from the retina. Wilcoxon rank sum test: $p < 0.0001$.

(**G**) Experimental timeline for primary murine mixed glial cultures to obtain microglia and astrocyte-enriched cultures for protein analysis. P0-P3: postnatal day 0 to 3. 4-OHT: 4-hydroxytamoxifen. Next: IBA1-immunostained (magenta) mixed glial culture at day 10 prior to trypsinization. Scale bar: 50 µm.

(**H**) Western blot analysis of UCP2 protein expression (left, top), β-actin loading control (left, bottom), IBA1 microglial marker (right, top), or SDHA (Succinate dehydrogenase complex flavoprotein subunit A) mitochondrial loading control (right, bottom) in primary microglia of either *Cx3cr1*$^{CreERT2/+}$ or *Cx3cr1*$^{CreERT2/+}$/$UCP2^{fl/fl}$ with vehicle or 4-OHT (n = 1). kDa, kilodaltons.

(**I**) Quantification of UCP2 band intensity relative to β-actin loading control (left) or relative to SDHA (right) mitochondrial loading control.

* $p < 0.05$, *** $p < 0.001$. See **Table S9** for retina and cell numbers, statistical tests and corresponding data.

Figure S5

A *Principal component loadings*

	PC 1	PC 2
% Mitochondria Perinuclear	0.4915	0.0797
Median mitochondrial volume	-0.3809	0.5161
Number of mitochondria	0.2067	-0.5712
% Mitochondria volume/microglia	0.3184	0.6000
Sholl index	0.5110	0.1991
CD68 volume	0.4563	-0.0379

B *Mitochondrial content*

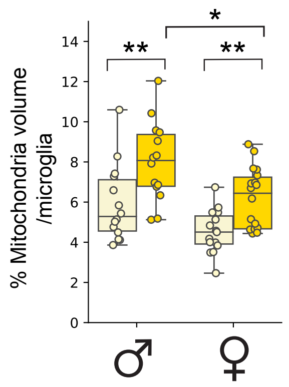

C *Mitochondrial connectivity*

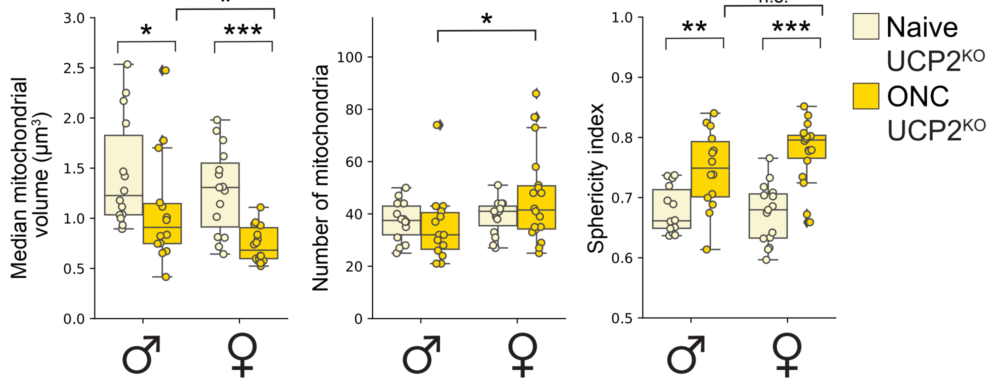

D *Cellular ROS in WT male and female*

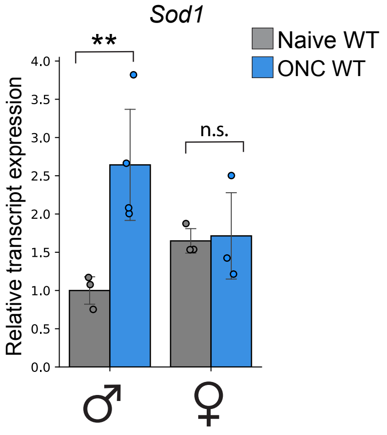

E *Mitochondrial ROS in WT ONC male and female*

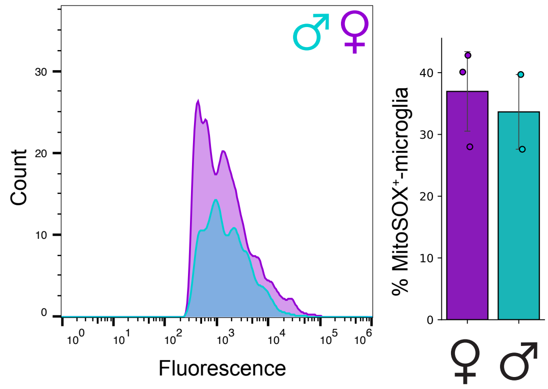

Figure S5. Sex-separated UCP2^{KO} mitochondrial parameters, Related to Figure 5

(**A**) Table of principal component (PC) loadings from **Figure 5**. Green highlight: Highest loading for PC2.

(**B**-**C**) Mitochondrial parameters. Boxplot minimum and maximum: InterQuartile Range (IQR) around median (center line). Whiskers: 1.5 IQRs. Black diamond: outliers outside 1.5 IQRs. Each overlaid point represents data point of a single microglia. Naïve UCP2^{KO}: tan. ONC UCP2^{KO}: gold.

(**B)** Percentage of mitochondrial volume per microglial volume. Kruskal-Wallis test: $p < 0.0001$. Selected Conover's post-hoc comparisons with Holm p-adjustment: Naïve ♂UCP2^{KO} *vs*. ONC ♂UCP2^{KO}, $p = 0.0076$; Naïve ♀UCP2^{KO} *vs*. ONC ♀UCP2^{KO}, $p = 0.0073$; ONC ♂UCP2^{KO} *vs*. ONC ♀UCP2^{KO}, $p = 0.0266$.

(**C**) Mitochondrial network connectivity determined by median mitochondrial volume (left, Kruskal-Wallis test: $p < 0.0001$. Selected Conover's post-hoc comparisons with Holm p-adjustment: Naïve ♂UCP2^{KO} *vs*. ONC ♂UCP2^{KO}, $p = 0.0348$; Naïve ♀UCP2^{KO} *vs*. ONC ♀UCP2^{KO}, $p < 0.0001$; ONC ♂UCP2^{KO} *vs*. ONC ♀UCP2^{KO}, $p = 0.0303$), number of organelles (center, Wilcoxon rank sum test: $p = 0.0383$) and mean sphericity (right, Kruskal-Wallis test: $p < 0.0001$. Selected Conover's post-hoc comparisons with Holm p-adjustment: Naïve ♂UCP2^{KO} *vs*. ONC ♂UCP2^{KO}, $p = 0.0029$; Naïve ♀UCP2^{KO} *vs*. ONC ♀UCP2^{KO}, $p < 0.0001$; ONC ♂UCP2^{KO} *vs*. ONC ♀UCP2^{KO}, $p = 0.2323$).

(**D**) Bar plot depicting relative *Sod1* transcript expression from FACSed mito-Dendra2^{+} microglia in naïve WT and ONC WT male and female microglia. Transcript expression relative to male naïve WT. Kruskal-Wallis test: $p = 0.0269$. Selected Conover's post-hoc comparisons with Holm p-adjustment: ♂WT Naïve *vs*. ♂WT ONC: $p = 0.0026$, ♀WT Naïve *vs*. ♀WT ONC: $p = 0.8554$.

(**E**) Frequency plot of MitoSOX fluorescence from FACSed mito-Dendra2^{+} retinal microglia for WT ONC male (turquoise) and female (purple). Corresponding bar plot of percentage of microglia that are MitoSOX^{+}.

** $p < 0.01$, *** $p < 0.001$, $^{n.s.}$ $p > 0.05$: not significant. See **Table S10** for retina and cell numbers, statistical tests, other post-hoc comparisons and corresponding data.

Table S11. Primer sequences, Related to STAR Methods.

Gene	Primer	Sequence	Product length
**GAPDH**	FW	ACAGCAACTCCCACTCTTC	131 bp
	REV	CATTGTCATACCAGGAAATGAGC	
**IBA1**	FW	ATGAGGATCTGCCGTCCAAAC	114 bp
	REV	CAAGTTTCTCCAGCATTCGCTTC	
**UCP2**	FW	AAGTGTTTCGTCTCCCAGCC	188 bp
	REV	GGGACCTTCAATCGGCAAGA	
**CD68**	FW	CCTCTGTTCCTTGGGCTATAAG	105 bp
	REV	ATTGAGGAAGGAACTGGTGTAG	
**SOD1**	FW	CACTTCGAGCAGAAGGCAAGC	186 bp
	REV	ATGCCTCTCTTCATCCGCCG	
